# Supplementary material for: A fault-intrusion-tolerant system and deadline-aware algorithm for scheduling scientific workflow in the cloud
Source: PeerJ Comput Sci. 2021 Nov 2;7:e747. doi: 10.7717/peerj-cs.747 (PMC8576554; doi:10.7717/peerj-cs.747)
Supplement: Supplemental Information 6 [file peerj-cs-07-747-s006.zip › Myproject1.1/docs/org/cloudbus/cloudsim/network/datacenter/NetDatacenterBroker.html]

NetDatacenterBroker


JavaScript is disabled on your browser.


- Overview
- Package
- Class
- Use
- Tree
- Deprecated
- Index
- Help

- Prev Class
- Next Class

- Frames
- No Frames

- All Classes

- Summary:
- Nested |
- Field |
- Constr |
- Method

- Detail:
- Field |
- Constr |
- Method


org.cloudbus.cloudsim.network.datacenter

## Class NetDatacenterBroker

- java.lang.Object
- - org.cloudbus.cloudsim.core.SimEntity
  - - org.cloudbus.cloudsim.network.datacenter.NetDatacenterBroker

- All Implemented Interfaces:
  :   java.lang.Cloneable

  ---

    

  ```
  public class NetDatacenterBroker
  extends SimEntity
  ```

  NetDatacentreBroker represents a broker acting on behalf of Datacenter provider. It hides VM
  management, as vm creation, submission of cloudlets to this VMs and destruction of VMs. NOTE- It
  is an example only. It work on behalf of a provider not for users. One has to implement
  interaction with user broker to this broker.

  Since:
  :   CloudSim Toolkit 3.0

- - ### Field Summary

    Fields

    | Modifier and Type | Field and Description |
    | --- | --- |
    | `static int` | `cachedcloudlet` |
    | `boolean` | `createvmflag` |
    | `static NetworkDatacenter` | `linkDC` |

    - ### Fields inherited from class org.cloudbus.cloudsim.core.SimEntity

      `FINISHED, HOLDING, RUNNABLE, WAITING`
  - ### Constructor Summary

    Constructors

    Constructor and Description

    `NetDatacenterBroker(java.lang.String name)`

    Created a new DatacenterBroker object.
  - ### Method Summary

    Methods

    | Modifier and Type | Method and Description |
    | --- | --- |
    | `protected void` | `clearDatacenters()` Submit cloudlets to the created VMs. |
    | `protected void` | `createVmsInDatacenterBase(int datacenterId)` Create the virtual machines in a datacenter and submit/schedule cloudlets to them. |
    | `<T extends AppCloudlet>  java.util.List<T>` | `getAppCloudletList()` |
    | `<T extends NetworkCloudlet>  java.util.List<T>` | `getCloudletList()` Gets the cloudlet list. |
    | `<T extends Cloudlet>  java.util.List<T>` | `getCloudletReceivedList()` Gets the cloudlet received list. |
    | `<T extends Cloudlet>  java.util.List<T>` | `getCloudletSubmittedList()` Gets the cloudlet submitted list. |
    | `protected java.util.Map<java.lang.Integer,DatacenterCharacteristics>` | `getDatacenterCharacteristicsList()` Gets the datacenter characteristics list. |
    | `protected java.util.List<java.lang.Integer>` | `getDatacenterIdsList()` Gets the datacenter ids list. |
    | `protected java.util.List<java.lang.Integer>` | `getDatacenterRequestedIdsList()` Gets the datacenter requested ids list. |
    | `<T extends Vm>  java.util.List<T>` | `getVmList()` Gets the vm list. |
    | `protected int` | `getVmsAcks()` Gets the vms acks. |
    | `<T extends Vm>  java.util.List<T>` | `getVmsCreatedList()` Gets the vm list. |
    | `protected int` | `getVmsDestroyed()` Gets the vms destroyed. |
    | `protected int` | `getVmsRequested()` Gets the vms requested. |
    | `protected java.util.Map<java.lang.Integer,java.lang.Integer>` | `getVmsToDatacentersMap()` Gets the vms to datacenters map. |
    | `protected void` | `incrementVmsAcks()` Increment vms acks. |
    | `protected void` | `processCloudletReturn(SimEvent ev)` Process a cloudlet return event. |
    | `void` | `processEvent(SimEvent ev)` Processes events available for this Broker. |
    | `protected void` | `processOtherEvent(SimEvent ev)` Overrides this method when making a new and different type of Broker. |
    | `protected void` | `processResourceCharacteristics(SimEvent ev)` Process the return of a request for the characteristics of a PowerDatacenter. |
    | `protected void` | `processResourceCharacteristicsRequest(SimEvent ev)` Process a request for the characteristics of a PowerDatacenter. |
    | `<T extends AppCloudlet>  void` | `setAppCloudletList(java.util.List<T> appCloudletList)` |
    | `protected <T extends NetworkCloudlet>  void` | `setCloudletList(java.util.List<T> cloudletList)` Sets the cloudlet list. |
    | `protected <T extends Cloudlet>  void` | `setCloudletReceivedList(java.util.List<T> cloudletReceivedList)` Sets the cloudlet received list. |
    | `protected <T extends Cloudlet>  void` | `setCloudletSubmittedList(java.util.List<T> cloudletSubmittedList)` Sets the cloudlet submitted list. |
    | `protected void` | `setDatacenterCharacteristicsList(java.util.Map<java.lang.Integer,DatacenterCharacteristics> datacenterCharacteristicsList)` Sets the datacenter characteristics list. |
    | `protected void` | `setDatacenterIdsList(java.util.List<java.lang.Integer> datacenterIdsList)` Sets the datacenter ids list. |
    | `protected void` | `setDatacenterRequestedIdsList(java.util.List<java.lang.Integer> datacenterRequestedIdsList)` Sets the datacenter requested ids list. |
    | `void` | `setLinkDC(NetworkDatacenter alinkDC)` |
    | `protected <T extends Vm>  void` | `setVmList(java.util.List<T> vmList)` Sets the vm list. |
    | `protected void` | `setVmsAcks(int vmsAcks)` Sets the vms acks. |
    | `protected <T extends Vm>  void` | `setVmsCreatedList(java.util.List<T> vmsCreatedList)` Sets the vm list. |
    | `protected void` | `setVmsDestroyed(int vmsDestroyed)` Sets the vms destroyed. |
    | `protected void` | `setVmsRequested(int vmsRequested)` Sets the vms requested. |
    | `protected void` | `setVmsToDatacentersMap(java.util.Map<java.lang.Integer,java.lang.Integer> vmsToDatacentersMap)` Sets the vms to datacenters map. |
    | `void` | `shutdownEntity()` This method is invoked by the `Simulation` before the simulation finishes. |
    | `void` | `startEntity()` This method is invoked by the `Simulation` class when the simulation is started. |
    | `void` | `submitCloudletList(java.util.List<? extends NetworkCloudlet> list)` This method is used to send to the broker the list of cloudlets. |
    | `void` | `submitVmList(java.util.List<? extends Vm> list)` This method is used to send to the broker the list with virtual machines that must be created. |

    - ### Methods inherited from class org.cloudbus.cloudsim.core.SimEntity

      `cancelEvent, clone, getEventBuffer, getId, getName, getNextEvent, getNextEvent, getState, numEventsWaiting, numEventsWaiting, pause, run, schedule, schedule, schedule, schedule, scheduleFirst, scheduleFirst, scheduleFirst, scheduleFirst, scheduleFirstNow, scheduleFirstNow, scheduleFirstNow, scheduleFirstNow, scheduleNow, scheduleNow, scheduleNow, scheduleNow, selectEvent, send, send, send, send, sendNow, sendNow, sendNow, sendNow, setEventBuffer, setId, setState, waitForEvent`
    - ### Methods inherited from class java.lang.Object

      `equals, finalize, getClass, hashCode, notify, notifyAll, toString, wait, wait, wait`

- - ### Field Detail


    - #### linkDC

      ```
      public static NetworkDatacenter linkDC
      ```


    - #### createvmflag

      ```
      public boolean createvmflag
      ```


    - #### cachedcloudlet

      ```
      public static int cachedcloudlet
      ```
  - ### Constructor Detail


    - #### NetDatacenterBroker

      ```
      public NetDatacenterBroker(java.lang.String name)
                          throws java.lang.Exception
      ```

      Created a new DatacenterBroker object.

      Parameters:
      :   `name` - name to be associated with this entity (as required by Sim\_entity class from
          simjava package)

      Throws:
      :   `java.lang.Exception` - the exception
  - ### Method Detail


    - #### submitVmList

      ```
      public void submitVmList(java.util.List<? extends Vm> list)
      ```

      This method is used to send to the broker the list with virtual machines that must be
      created.

      Parameters:
      :   `list` - the list


    - #### submitCloudletList

      ```
      public void submitCloudletList(java.util.List<? extends NetworkCloudlet> list)
      ```

      This method is used to send to the broker the list of cloudlets.

      Parameters:
      :   `list` - the list


    - #### setLinkDC

      ```
      public void setLinkDC(NetworkDatacenter alinkDC)
      ```


    - #### processEvent

      ```
      public void processEvent(SimEvent ev)
      ```

      Processes events available for this Broker.

      **Specified by:**
      :   `processEvent` in class `SimEntity`

      Parameters:
      :   `ev` - a SimEvent object


    - #### processResourceCharacteristics

      ```
      protected void processResourceCharacteristics(SimEvent ev)
      ```

      Process the return of a request for the characteristics of a PowerDatacenter.

      Parameters:
      :   `ev` - a SimEvent object


    - #### processResourceCharacteristicsRequest

      ```
      protected void processResourceCharacteristicsRequest(SimEvent ev)
      ```

      Process a request for the characteristics of a PowerDatacenter.

      Parameters:
      :   `ev` - a SimEvent object


    - #### processCloudletReturn

      ```
      protected void processCloudletReturn(SimEvent ev)
      ```

      Process a cloudlet return event.

      Parameters:
      :   `ev` - a SimEvent object


    - #### processOtherEvent

      ```
      protected void processOtherEvent(SimEvent ev)
      ```

      Overrides this method when making a new and different type of Broker. This method is called
      by `#body()` for incoming unknown tags.

      Parameters:
      :   `ev` - a SimEvent object


    - #### createVmsInDatacenterBase

      ```
      protected void createVmsInDatacenterBase(int datacenterId)
      ```

      Create the virtual machines in a datacenter and submit/schedule cloudlets to them.

      Parameters:
      :   `datacenterId` - Id of the chosen PowerDatacenter


    - #### clearDatacenters

      ```
      protected void clearDatacenters()
      ```

      Submit cloudlets to the created VMs.


    - #### shutdownEntity

      ```
      public void shutdownEntity()
      ```

      **Description copied from class: `SimEntity`**

      This method is invoked by the `Simulation` before the simulation finishes. If you want
      to save data in log files this is the method in which the corresponding code would be placed.

      **Specified by:**
      :   `shutdownEntity` in class `SimEntity`


    - #### startEntity

      ```
      public void startEntity()
      ```

      **Description copied from class: `SimEntity`**

      This method is invoked by the `Simulation` class when the simulation is started. This
      method should be responsible for starting the entity up.

      **Specified by:**
      :   `startEntity` in class `SimEntity`


    - #### getVmList

      ```
      public <T extends Vm> java.util.List<T> getVmList()
      ```

      Gets the vm list.

      Type Parameters:
      :   `T` - the generic type

      Returns:
      :   the vm list


    - #### setVmList

      ```
      protected <T extends Vm> void setVmList(java.util.List<T> vmList)
      ```

      Sets the vm list.

      Type Parameters:
      :   `T` - the generic type

      Parameters:
      :   `vmList` - the new vm list


    - #### getCloudletList

      ```
      public <T extends NetworkCloudlet> java.util.List<T> getCloudletList()
      ```

      Gets the cloudlet list.

      Type Parameters:
      :   `T` - the generic type

      Returns:
      :   the cloudlet list


    - #### setCloudletList

      ```
      protected <T extends NetworkCloudlet> void setCloudletList(java.util.List<T> cloudletList)
      ```

      Sets the cloudlet list.

      Type Parameters:
      :   `T` - the generic type

      Parameters:
      :   `cloudletList` - the new cloudlet list


    - #### getAppCloudletList

      ```
      public <T extends AppCloudlet> java.util.List<T> getAppCloudletList()
      ```


    - #### setAppCloudletList

      ```
      public <T extends AppCloudlet> void setAppCloudletList(java.util.List<T> appCloudletList)
      ```


    - #### getCloudletSubmittedList

      ```
      public <T extends Cloudlet> java.util.List<T> getCloudletSubmittedList()
      ```

      Gets the cloudlet submitted list.

      Type Parameters:
      :   `T` - the generic type

      Returns:
      :   the cloudlet submitted list


    - #### setCloudletSubmittedList

      ```
      protected <T extends Cloudlet> void setCloudletSubmittedList(java.util.List<T> cloudletSubmittedList)
      ```

      Sets the cloudlet submitted list.

      Type Parameters:
      :   `T` - the generic type

      Parameters:
      :   `cloudletSubmittedList` - the new cloudlet submitted list


    - #### getCloudletReceivedList

      ```
      public <T extends Cloudlet> java.util.List<T> getCloudletReceivedList()
      ```

      Gets the cloudlet received list.

      Type Parameters:
      :   `T` - the generic type

      Returns:
      :   the cloudlet received list


    - #### setCloudletReceivedList

      ```
      protected <T extends Cloudlet> void setCloudletReceivedList(java.util.List<T> cloudletReceivedList)
      ```

      Sets the cloudlet received list.

      Type Parameters:
      :   `T` - the generic type

      Parameters:
      :   `cloudletReceivedList` - the new cloudlet received list


    - #### getVmsCreatedList

      ```
      public <T extends Vm> java.util.List<T> getVmsCreatedList()
      ```

      Gets the vm list.

      Type Parameters:
      :   `T` - the generic type

      Returns:
      :   the vm list


    - #### setVmsCreatedList

      ```
      protected <T extends Vm> void setVmsCreatedList(java.util.List<T> vmsCreatedList)
      ```

      Sets the vm list.

      Type Parameters:
      :   `T` - the generic type

      Parameters:
      :   `vmsCreatedList` - the vms created list


    - #### getVmsRequested

      ```
      protected int getVmsRequested()
      ```

      Gets the vms requested.

      Returns:
      :   the vms requested


    - #### setVmsRequested

      ```
      protected void setVmsRequested(int vmsRequested)
      ```

      Sets the vms requested.

      Parameters:
      :   `vmsRequested` - the new vms requested


    - #### getVmsAcks

      ```
      protected int getVmsAcks()
      ```

      Gets the vms acks.

      Returns:
      :   the vms acks


    - #### setVmsAcks

      ```
      protected void setVmsAcks(int vmsAcks)
      ```

      Sets the vms acks.

      Parameters:
      :   `vmsAcks` - the new vms acks


    - #### incrementVmsAcks

      ```
      protected void incrementVmsAcks()
      ```

      Increment vms acks.


    - #### getVmsDestroyed

      ```
      protected int getVmsDestroyed()
      ```

      Gets the vms destroyed.

      Returns:
      :   the vms destroyed


    - #### setVmsDestroyed

      ```
      protected void setVmsDestroyed(int vmsDestroyed)
      ```

      Sets the vms destroyed.

      Parameters:
      :   `vmsDestroyed` - the new vms destroyed


    - #### getDatacenterIdsList

      ```
      protected java.util.List<java.lang.Integer> getDatacenterIdsList()
      ```

      Gets the datacenter ids list.

      Returns:
      :   the datacenter ids list


    - #### setDatacenterIdsList

      ```
      protected void setDatacenterIdsList(java.util.List<java.lang.Integer> datacenterIdsList)
      ```

      Sets the datacenter ids list.

      Parameters:
      :   `datacenterIdsList` - the new datacenter ids list


    - #### getVmsToDatacentersMap

      ```
      protected java.util.Map<java.lang.Integer,java.lang.Integer> getVmsToDatacentersMap()
      ```

      Gets the vms to datacenters map.

      Returns:
      :   the vms to datacenters map


    - #### setVmsToDatacentersMap

      ```
      protected void setVmsToDatacentersMap(java.util.Map<java.lang.Integer,java.lang.Integer> vmsToDatacentersMap)
      ```

      Sets the vms to datacenters map.

      Parameters:
      :   `vmsToDatacentersMap` - the vms to datacenters map


    - #### getDatacenterCharacteristicsList

      ```
      protected java.util.Map<java.lang.Integer,DatacenterCharacteristics> getDatacenterCharacteristicsList()
      ```

      Gets the datacenter characteristics list.

      Returns:
      :   the datacenter characteristics list


    - #### setDatacenterCharacteristicsList

      ```
      protected void setDatacenterCharacteristicsList(java.util.Map<java.lang.Integer,DatacenterCharacteristics> datacenterCharacteristicsList)
      ```

      Sets the datacenter characteristics list.

      Parameters:
      :   `datacenterCharacteristicsList` - the datacenter characteristics list


    - #### getDatacenterRequestedIdsList

      ```
      protected java.util.List<java.lang.Integer> getDatacenterRequestedIdsList()
      ```

      Gets the datacenter requested ids list.

      Returns:
      :   the datacenter requested ids list


    - #### setDatacenterRequestedIdsList

      ```
      protected void setDatacenterRequestedIdsList(java.util.List<java.lang.Integer> datacenterRequestedIdsList)
      ```

      Sets the datacenter requested ids list.

      Parameters:
      :   `datacenterRequestedIdsList` - the new datacenter requested ids list


- Overview
- Package
- Class
- Use
- Tree
- Deprecated
- Index
- Help

- Prev Class
- Next Class

- Frames
- No Frames

- All Classes

- Summary:
- Nested |
- Field |
- Constr |
- Method

- Detail:
- Field |
- Constr |
- Method
